# The Concordance between Patients’ Renal Replacement Therapy Choice and Definitive Modality: Is It a Utopia?

**DOI:** 10.1371/journal.pone.0138811

**Published:** 2015-10-14

**Authors:** Mario Prieto-Velasco, Pedro Quiros, Cesar Remon, Spanish Group for the Implementation of a Shared Decision Making Process for RRT Choice with Patient Decision Aid Tools

**Affiliations:** 1 Department of Nephrology, Complejo Asistencial Universitario de León, León, Spain; 2 Department of Nephrology, Hospital Universitario de Puerto Real, Puerto Real, Spain; University of Sao Paulo Medical School, BRAZIL

## Abstract

**Introduction:**

It is desirable for patients to play active roles in the choice of renal replacement therapy (RRT). Patient decision aid tools (PDAs) have been developed to allow the patients to choose the option best suited to their individual needs.

**Material and Methods:**

An observational, prospective registry was conducted in 26 Spanish hospitals between September 2010 and May 2012. The results of the patients’ choice and the definitive RRT modality were registered through the progressive implementation of an Education Process (EP) with PDAs designed to help Chronic Kidney Disease (CKD) patients choose RRT.

**Results:**

Patients included in this study: 1044. Of these, 569 patients used PDAs and had made a definitive choice by the end of registration. A total of 88.4% of patients chose dialysis [43% hemodialysis (HD) and 45% peritoneal dialysis (PD)] 3.2% preemptive living-donor transplant (TX), and 8.4% conservative treatment (CT). A total of 399 patients began RRT during this period. The distribution was 93.4% dialysis (53.6% HD; 40% PD), 1.3% preemptive TX and 5.3% CT. The patients who followed the EP changed their mind significantly less often [kappa value of 0.91 (95% CI, 0.86–0.95)] than those who did not follow it, despite starting unplanned treatment [kappa value of 0.85 (95% CI, 0.75–0.95]. A higher agreement between the final choice and a definitive treatment was achieved by the EP and planned patients [kappa value of 0.93 (95% CI, 0.89–0.98)]. Those who did not go through the EP had a much lower index of choosing PD and changed their decision more frequently when starting definitive treatment [kappa value of 0.73 (95% CI, 0.55–0.91)].

**Conclusions:**

Free choice, assisted by PDAs, leads to a 50/50 distribution of PD and HD choice and an increase in TX choice. The use of PDAs, even with an unplanned start, achieved a high level of concordance between the chosen and definitive modality.

## Introduction

In medical practice, situations arise in which there are two or more treatment options that have shown equivalent results but involve major differences in the effects they will have on various important personal aspects (e.g., lifestyle, body image, and family involvement). In these situations, it is increasingly suggested that patients choose the treatment option that adapts most closely to their values and lifestyle. This approach is upheld by scientific societies and legislation. Renal Replacement Treatment (RRT) is one such situation [1–2].

There are many aspects of the current decision making process that have been negatively evaluated by patients (such as time devoted, clarity of explanations, and availability of healthcare professionals (HCP) to answer questions) [3]. A significant percentage of patients state that they were not informed about treatment options, and for half of the patients, the information received was insufficient to allow them to understand the differences between therapeutic alternatives [3–7]. Additionally, there is scarce published data in this aspect of RRT; it is unknown to what degree patient participation is implemented during the RRT modality choice in the real world, and there is a lack of published guidelines on how to efficiently perform this process. However, some relevant papers have been recently published [8–10].

Although there are small studies that have shown a higher choice of home treatment options when patient education is provided, most of the dialysis and transplantation registries do not show a balanced distribution in the incidence of the different dialysis modalities [11]. Similarly, preemptive transplantation (TX), despite being the most efficient modality, is not the most frequently chosen option in many countries [11]. Thus, in-center Hemodialysis (HD) continues to be the most commonly selected option for starting RRT [12–14]. Frequently, an unscheduled start of dialysis with urgent HD determines the permanence of this modality [4,15].

However, little is known about patients’ psychosocial and cognitive conditions as contributing factors to dialysis modality decision-making. In this regard, the Choice of Renal Replacement Therapy (CORETH), a multicenter study that is being conducted in Germany, aims to examine these conditions with regard to their impact on the choice [16].

In such a context and in addition to legal compliance[1] and respect for patients’ rights and values, a structured decision-making process aims for a better adaptation of the treatment to the patient, a better preparation for TX, and a more balanced choice of home modalities [4,14–24] and conservative treatment (CT).

Patient Decision Aid Tools (PDAs) have been developed to help patients carefully consider the treatment options and to understand their peculiarities and the impact that the elected option will have on their lives.

We present the results of the implementation of a decision-making process based on these PDAs which have been designed specifically for people with renal failure.

## Subjects and Methods

An observational, prospective, multicenter registry was conducted in 26 Spanish hospitals to determine the impact of a structured Education Process (EP) with PDAs for RRT patients’ choice.

The authors of this paper represent a multicenter group of healthcare professionals from the Chronic Kidney Disease (CKD) clinics of the above mentioned hospitals (both nephrologists and nurses). Regarding the interaction that the authors had with the patients, they are themselves healthcare professionals in charge of their own CKD units.

The education process was not implemented for the sake of this study. It was developed several years previously, and this manuscript only describes the activity that was previously underway. Furthermore, this education process remains ongoing in these and other hospitals nationwide. To compile the data from each of the centers, a simple Excel sheet for registering the de-identified patients’ information was developed, and the patients’ personal data were never registered, to comply with the Spanish Law.

Patient data were extracted and provided to the research team as an Excel file by the members of the Spanish Group for the Implementation of a Shared Decision Making Process for RRT Choice with Patient Decision Aids that are included in the acknowledgments section of this manuscript and who are the healthcare professionals in charge of the normal patients’ care in each of their own CKD units. All of the participating hospitals permitted this study to be conducted using their patient data. When analyzing the data, the authors did not have access to any identifying patient information and the patients involved in this initiative explicitly stated their verbal informed consent to use their de-identified information for this purpose, which was registered in their medical record. The following data were extracted: sex; age; whether the patient was known by the nephrology department when treatment was initiated; the date of first dialysis; the first dialysis modality; vascular or peritoneal access; whether the educational process was given; the treatment modality chosen by the patient; the definitive treatment modality after the start of the treatment; the definitive vascular or peritoneal access; the date of the first patient visit of the educational process; the glomerular filtrate rate at the time of the first patient visit; the dates for the second, third, fourth and fifth visits (if needed); the decision making status at visits 2, 3, 4 and 5 (if needed); the patient's initial choice; the patient's final choice; and the glomerular filtrate rate at the date of the patient’s choice.

Regarding the EP, in Spain there is Law 41/2002, regulatory base of patient autonomy and of rights and obligations with regard to clinical information and documentation. B.O.E. number 274, November 15, 2002. This law covers the rights of patients to receive information on therapeutic alternatives and choose among them. Verbal consent is valid under the Spanish legislation for educational/information processes, and verbal consent is always recorded in the patient’s medical record. Once the educational process and the information on therapeutic alternatives have been provided, the patient (or the legal representative) signs the required informed consent for the information provided and the treatment choice. For educational and/or informative interventions ethics committees’ approval is not applicable in Spain; nevertheless, this work has been submitted to the committee of one of the participating hospitals, and it has been exempted from approval.

The implementation of the EP in each of the hospitals progressively started (September 2010-May 2012). Once the process was in place in each of the hospitals, all CKD patients with an indication for an RRT or CT were registered. Patients who required an unplanned start of dialysis during the same period were also independently registered, whether the EP was provided before or after starting dialysis.

Given the available data, and although it is not a globally agreed definition, unplanned patients have been considered in this study as those who started dialysis who were previously unknown by the Nephrology Unit, and/or who started it in an urgent situation, and/or without permanent dialysis access, and/or without having gone through the EP, independently of the referral time.

### Education Process

In our setting, patients, their relatives, and a team of health professionals participate in the EP, generally involving a nephrologist and an education-skilled nurse (both funded by the National Healthcare System with no special budget allocated to the CKD education process). Others can also participate, such as social workers, psychologists and mentor patients.

The EP is structured into 4 sequential phases that were established based on an elicit-provide-elicit process: elicit patients’ values and preferences (values phase), provide information (informational phase), elicit what they think about the information received and how it empowers them (deliberation and question-and-answer phase), and enable them to actively make a decision.

The first phase (values phase) aims for patients to identify their values, which are understood as their preferences, their lifestyle, the aspects they wish to maintain in their lives and the aspects they absolutely reject. The EP is based on a respect of these values, regardless of whether they agree with the rest of the team’s values. It is performed in the outpatient setting and lasts approximately 40 minutes.

The second phase (informational phase) consists of providing the patient and their relatives with formal, balanced and realistic information about the CKD, CT and RRT modalities (continuous ambulatory peritoneal dialysis (CAPD), automated peritoneal dialysis (APD), in-center HD, home HD, and TX (live or deceased donor). In our environment, we define CT as the treatment of the complications derived from CKD stage V, excluding RRT. This phase starts at home with specific educational tools and is followed by the second patient visit to the clinic, which lasts approximately 1 hour (although can be divided into 2 shorter sessions).

Next is the deliberation and question-and-answer phase. Patients and their relatives are guided to reflect on their choices and resolve any uncertainty that might have arisen. It consists of a variable number of visits (0 to 3) lasting 15–20 minutes each, until a final choice is made.

**Fig 1:**
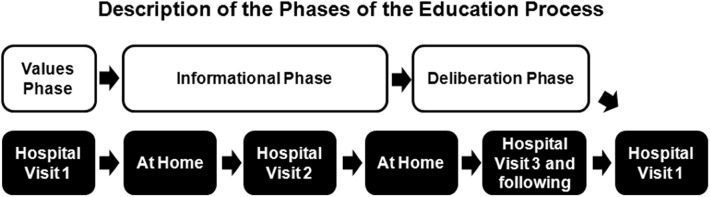
Description of the Phases of the Education Process.

The final phase is the decision-making phase. Patients confirm their treatment choice and sign the informed consent forms together with the nephrologist, who records the definitive decision and starts the RRT preparation phase (Fig 1).

After the patients have chosen the definitive modality (and the dialysis access has been performed for those choosing dialysis treatments), they are referred to the corresponding medical units to be followed-up by the new healthcare teams.

The supporting media used during each of the phases were 1) Electronic format: decision-making software (questionnaire about patients’ preferences), questionnaire schedule of a work day and a normal weekend, DVD; 2) Paper format: flipchart, values cards, abbreviated brochure about dialysis modalities, informational brochure for relatives and/or friends, expanded brochure about CKD, RRT modalities, CT and TX, leaflet about social assistance benefits; and 3) Forms: advantages and points to consider about dialysis modalities and situation form regarding decision making (S1 File. Components of the Decision Aid Tools and Activities).

All of the material comprises the PDAs that had been specifically designed to cover two objectives of the EP: to progressively provide information and to guide patients to identify their personal values and important lifestyle aspects and preferences, facilitating the deliberation and ultimately the choice. Examples of patients’ values and uncertainties are: how will each of the treatment options interfere with my freedom and autonomy?; what the impact be on my family?; how would they affect my day-time schedule?; how often will I have to visit the hospital?; how much time will it take to go to the dialysis center?; will I be able to maintain my job?; how will the treatment influence my social life?; and, will I need to be punctured every time?

### Statistical Analysis

The main research question was whether the structured method of education with PDAs in the CKD stage influences the initial RRT choice and the definitive treatment modality. To test this hypothesis, we compared patients that pass through the EP vs. those that do not; patients with a planned start vs. patients with an urgent dialysis start; and the type of vascular or peritoneal access with which patients start dialysis according to the above variables.

Statistical analysis was performed using measurements of central tendency and dispersion (median and interquartile intervals Q1 –Q3 or, when appropriate, mean ± standard deviation) for quantitative variables and frequencies for qualitative variables. For inferential statistics, the data were compared using the χ2test, according to the types of variables, with the use of contingency tables for causal relationships. Statistical significance was defined as an alpha error value (p)<0.05.

Concordance between the final choice and the definitive treatment was examined by a calculation of the accuracy and Cohen’s kappa (Cohen, 1960). The kappa statistic is represented by the extra amount of agreement observed after a consideration of the effect of chance over the maximum amount of such agreement that could theoretically occur (Brennan and Silman, 1992).

## Results and Discussion

### Patient Characteristics

One thousand and forty four patients from 26 hospitals were registered. Of these 890 (86.2%) passed through the EP and 569 (54.5%) had made a definitive choice by the end of registration. Of the total registered population, 399 (38.2%) initiated treatment with the definitive modality. The characteristics of the patients are listed in Table 1.

**Table 1:** Patient Characteristics. The values are expressed as the median and interquartile intervals Q1 –Q3.

Total number of registered patients	1044				
Number of patients by center	34 [13.8–57.5]				
Number of participating centers	26				
Sex	M 59.9%; F 40.1%				
Patients’ age at the beginning of the CKD monitoring (years)	67.2 [56.3–76.5]				
Patients’ age at the beginning of the EP	66.5 [55.2–76.5]				
Patients’ age of those who began RRT	65.5 [52.3–75.0]				
Total number of patients with a final choice	63.2%				
GFR at the start of the education process (ml/min)	16.3 [13.0–21.0]				
GFR at the end of the decision-making phase (ml/min)	13.0 [10.0–17.7]				
Educated patients	86.2%				
Average number of patient visits until the final choice	2.6±0.8				
Time until the final choice (months)	1.8 [0.7–3.8]				
Patients who started RRT	38.2%				
Planned	54.4%				
Unplanned	45.6%				
First dialysis access	All	Educated	Non-educated	Planned	Unplanned
Temporary vascular access	19.1%	18.1%	23.9%	0%	36.5%
Permanent vascular catheter	17.8%	16.1%	25.4%	9.9%	24.8%
Native arteriovenous fistula	32.6%	28.0%	46.3%	39.4%	29.9%
Peritoneal catheter	30.5%	37.8%	4.5%	50.7%	8.8%
Total	100%	100%	100%	100%	100%

The distribution of patients by CKD stage was 1.1% in Stage 3, 36.7% in Stage 4 and 62.3% in Stage 5.

### Choice and Treatment Initiation

**Fig 2:**
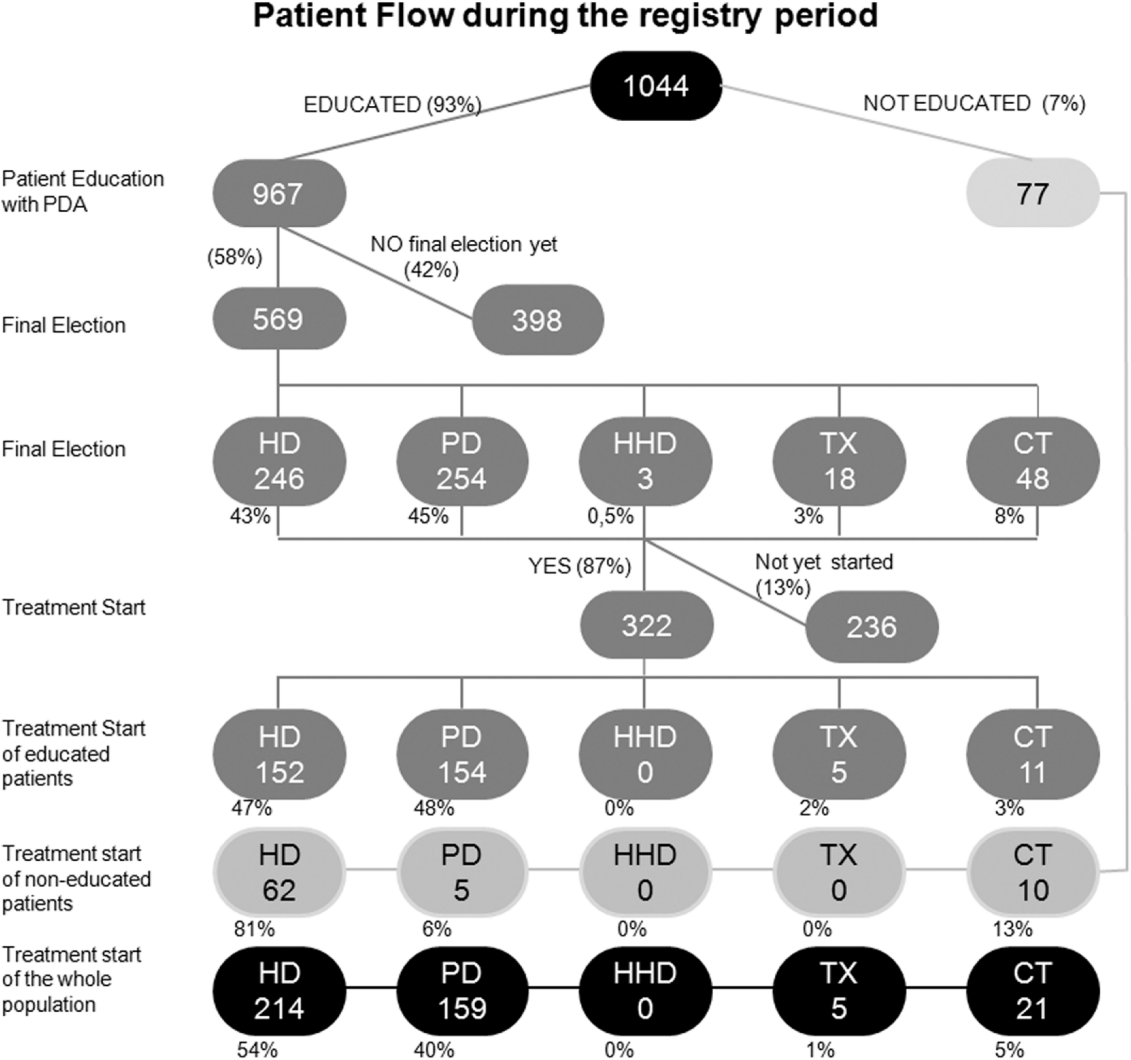
Patient Flow during the registry period. Abbreviations: CT, conservative treatment; HD, in-center hemodialysis; HHD, home hemodialysis; PD, peritoneal dialysis; and TX, living-donor preemptive transplantation.

We registered 1044 patients who had either started the decision-making process for RRT choice or who needed to begin RRT without having gone through the EP. Fig 2 shows the patient flow and the distribution of the modality choices at the start of treatment.

Among the population registered, 967 patients started the EP process, and 569 patients had a definitive choice at the close of registration. Of them, 43% of patients chose HD, 45% of patients chose PD, 3.2% of patients chose preemptive living donor TX, and 8.4% of patients chose CT (Fig 2 and Table 2). On the other hand, 399 patients began treatment during the registration period (322 patients after the EP, and 77 patients without education). The final distribution of the modalities was 373 (94%) dialysis (54% HD and 40% PD), 5 (1%) preemptive TX and 21 (5%) CT (Table 2). Chi-square test was used to determine whether there were significant differences between the final choice and the definitive modality for each of the treatment options. A statistically significant difference was found for PD (p = 0.03), as well as for TX and CT (p = 0.05); no statistical significance was found for HD (p = 0.17). However, since not all the patients with a final choice had a definitive modality and vice versa, the concordance between chosen and definitive modality will be analyzed further on in this article in the subgroup of patients with both sets of data.

**Table 2:** Choice and Definitive Treatment Method.

	Final Choice		Definitive modality		
	n	%	n	%	p Value*
**HD**	246	43.3%	214	53.6%	0.17
**PD**	257	45.1%	159	39.8%	0.03
**TX**	18	3.2%	5	1.3%	0.05
**CT**	48	8.4%	21	5.3%	0.05
**Total**	569	100%	399	100%	

(*) p value shows the difference between Final Choice and Definitive modality.

Abbreviations: RRT, renal replacement therapy; HD, in-center hemodialysis; PD, peritoneal dialysis; TX, living-donor preemptive transplantation; and CT, conservative treatment.

The average number of patient visits to the hospital was 2.6 (ranging from 2 to 5 visits), and the median time until a final choice was 1.8 [0.7–3.8] months. By visit 2, 50.1% of the patients had already made a definitive decision expressing their willingness to maintain it. This percentage increased as the process evolved and was up to 74.7% by visit 3, 82.7% by visit 4 and 100% by visit 5. The duration of the process was lower among the patients choosing TX than for those who opted for PD, HD or CT (p = 0.37).

The average time (months) until the final choice varied according to the final choice and was 3.33±4.1 HD, 2.99±3.9 PD, 1.55±1.6 TX and 3.6±2.6 CT.

On average, the patients for which HD or CT were the definitive modalities were older than those on PD or TX. The mean age (years) of the patients per each of the definitive modalities was 65.5±14.2 HD, 56.4±15.6 PD, 47.4±12.3 TX and 82.5±6.2 CT.

### Relationship between RRT Choice and Education Process

More educated patients started PD than patients who did not participate in an EP (47.8% vs. 6.5% (Chi-square test p<0.001)) (Table 3). This situation was the opposite for those included in HD, in which the percentage of non-educated patients was significantly greater (80.5% vs. 47.2%, Chi-square test p<0.001). Therefore, the EP group showed a 50/50 distribution between PD and HD.

**Table 3:** Definitive Treatment Method According to Whether the Patients Followed an Education Process or not.

	Considering All Treatment Modalities			Only Considering Dialysis		
	Educated Patients	Non-Educated Patients	Total	Educated Patients	Non-Educated Patients	Total
	n (%)	n (%)	n (%)	n (%)	n (%)	n (%)
**HD**	152 (47.2%)	62 (80.5%) *	214 (53.6%)	152 (49.7%)	62 (92.5%) *	214 (57.4%)
**PD**	154 (47.8%)	5 (6.5%) *	159 (39.8%)	154 (50.3%)	5 (7.5%) *	159 (42.6%)
**TX**	5 (1.6%)	0 (0.0%) *	5 (1.3%)			
**CT**	11 (3.4%)	10 (13%) *	21 (5.3%)			
**Total**	322 (100%)	77 (100%)	399 (100%)	306 (100%)	67 (100%)	373 (100%)

* p<0.001 compared with Educated Patients.

Abbreviations: RRT, renal replacement treatments; CT, conservative treatment; HD, in-center hemodialysis; PD, peritoneal dialysis; and TX, living-donor preemptive transplantation.

A higher proportion of patients opted for CT in the non-educated group (3.4% vs. 13%). Finally, all patients who began treatment with preemptive TX were educated.

### The Relationship between the Planned Dialysis Initiation and the Education Process

Of the 373 patients who started dialysis, 45.6% patients initiated unplanned dialysis. Of the educated group (306 patients), 66.1% (202) started planned dialysis and the remaining 33.9% (104) of the patients started it unplanned. In contrast, of the 67 non-educated patients who started dialysis, 66 (98.5%) patients started dialysis unplanned, and 1 (1.5%) patient started dialysis planned (p<0.001).

Hemodialysis was the start option for 88 (83.8%) of the educated unplanned patients, and the remaining 17 (16.2%) patients started it on PD. However, their definitive method after recovering from the acute condition was HD in 56 (53.3%) patients and PD in 49 (46.7%) patients, showing a more balanced dialysis choice distribution closer to the distribution of the whole EP group (p<0.001).

In the non-educated group, the distribution of HD as the initial and definitive method remained high (96.1% and 92.4%) (p<0.001) compared with the educated group.

### Chosen and Definitive Modality

The concordance between the treatment chosen and the definitive treatment modality with and without EP was analyzed with Kappa statistics. Although 399 patients began treatment during the registration period, both sets of data were collected in only 375 patients. A ‘good’ kappa value (Altman, 1991) of 0.73 (95% CI, 0.55–0.91) was obtained between the final choice and definitive treatment in the non-EP group. However, the kappa value was ‘very good’ in the EP group, 0.91 (95% CI, 0.86–0.95) (Table 4).

**Table 4:** Concordance between the Final Choice and the Definitive Method with and without an Education Process.

	Final Choice		Definitive Modality Distribution per Each Final Choice				
			HD	PD	TX	CT	
		n	n	n	n	n	Kappa (95% CI)
**Non-educated patients**	* *						0.73 (0.55–0.91)
	HD	45	44	1			
	PD	9	4	4		1	
	CT	9	1			8	
	*Total*	*63*	*49*	*5*		*9*	
**Educated patients**	* *		** **	** **	** **	** **	0.91 (0.86–0.95)
	HD	137	134	3			
	PD	158	11	147			
	TX	4			4		
	CT	13	2			11	
	*Total*	*312*	*147*	*150*	*4*	*11*	

Abbreviations: CT, conservative treatment; HD, in-center hemodialysis; PD, peritoneal dialysis; and TX, living-donor preemptive transplantation.

Additionally, differences were analyzed within the EP and non-EP groups considering a planned and unplanned start separately (Table 5). The kappa values are the following: non-EP-Unplanned 0.73 (95% CI, 0.55–0.91), EP-Unplanned 0.85 (95% CI, 0.75–0.95), and EP-Planned 0.93 (95% CI, 0.89–0.98). In summary, compared with the non-EP group, the educated patients, even those starting in an unplanned way, showed the highest concordance levels with ‘very good’ kappa values obtained between the final choice and the definitive modality. Ultimately, fewer patients changed their decision when the choice was made after passing through the education process.

**Table 5:** Concordance between the Final Choice and the Definitive Method with and without an Education Process in patients with a planned or an unplanned start.

	Final Choice		Definitive Modality Distribution per Each Final Choice				
			HD	PD	TX	CT	
		n	n	n	n	n	Kappa (95% CI)
**Non-educated patients**	*** ***						0.73 (0.55–0.91)
** **	***Unplanned start***						
	HD	44	43	1			
	PD	9	4	4		1	
	CT	9	1			8	
	*Total*	*62*	*48*	*5*	* *	*9*	
	* *	* *	* *			* *	-
	***Planned start***		** **			** **	
	HD	1	1				
	*Total*	*1*	*1*	* *	* *	* *	
**Educated patients**	* *		** **	** **	** **	** **	0.85 (0.75–0.95)
** **	***Unplanned start***		** **			** **	
	HD	46	45	1			
	PD	53	6	47			
	CT	2	1			1	
	*Total*	*101*	*52*	*48*	* *	*1*	
	*** ***	* *	* *			* *	0.93 (0.89–0.98)
	***Planned start***		** **			** **	
	HD	91	89	2			
	PD	105	5	100			
	TX	4			4		
	CT	11	1			10	
	*Total*	*211*	*95*	*102*	*4*	*10*	

Abbreviations: CT, conservative treatment; HD, in-center hemodialysis; PD, peritonealdialysis; and TX, living-donor preemptive transplantation.

## Discussion

To the best of our knowledge, this is one of the largest studies applying PDAs for RRT choice, and the results are derived from its application in 26 hospitals in various cultural, demographic and social environments in non-homogenous work settings, which confers appropriate applicability.

The median age of the population that underwent the EP was 67.2 [56.3–76.5] years, which is similar to the age of the population that currently starts treatment in our environment, according to the 2011 report of the European registry [11].

Our registry shows that 50% of the patients chose PD as the final treatment choice and the definitive treatment, provided the patients had gone through the EP. These results differ from the mean PD incidence in our country, which was 15%, according to the 2011 report of the Spanish registry (3% for TX and 82% for HD, without official data on CT choice), similar to that of neighboring countries [11].

Historically, the preemptive TX rate has been very low in Spain, most probably due to the significantly high cadaveric TX rate. Currently, living donor TX is being intensively promoted (including preemptive TX); however, the results of this registry, despite showing an increase in TX, remain disparate from the results of other countries. Likewise, home hemodialysis is scarcely chosen because this modality is available only in a few centers in Spain. Nevertheless, the results following the change in the patients’ education process are starting to become apparent in the latest regional registries data.

In terms of definitive treatment between EP and non-EP patients, the main difference was also the use of PD (50.3% vs. 7.5%). Multiple factors, both medical [25] and non-medical [16,26], probably played a role in this distribution. The fact that none of the non-EP patients began treatment with TX is also noteworthy.

However, comorbidities were not recorded, and the patients’ health literacy and numeracy level were also not registered. Nonetheless, the main duty of the educator is to adapt the information to the health literacy level of patients, caregivers and relatives. Additionally, for those patients with an impaired cognitive capacity, the family members and/or caregivers are those who participate in the EP process.

By means of the presented statistical analysis, it is difficult to draw conclusions about the direction of the relationships. However, assuring that the patients have access to all treatment modalities suitable for them, these results could support the idea that free choice does not really exist without education. When choices are offered without bias, the choice tends to a 50/50 split between HD and PD [6,25,27–28].

Patients choosing PD, HD or CT, compared with those choosing TX, needed a greater amount of time until the final decision, even though the difference was not statistically significant. Although we do not have additional data or a clear explanation for this result, patients choosing TX tend to be younger and active.

One of the aspects under continuous review regarding PDAs is how to quantify the quality of the decision. The IPDAS [29] defines “decision quality” as "the extent to which patients' eventual choices are consistent with their informed values and are actually implemented.” In this experience, at the end of the EP, the patients are asked to confirm that the decision is firm and that they have no willingness to change it (even though they can still change it at any time). In a study by Buck [12], 77% of the patients who chose PD ultimately started it, compared with 75.6% in a study by Goovaerts [13] and 48% in a study by Liebman [14]. In our opinion, the concordance between the final choice and definitive treatment is crucial for measuring the quality of a good shared decision-making process. Our experience contributes to previous knowledge that yields an excellent concordance of results. Although, non-EP show a good level of agreement between the final choice and the definitive modality, EP patients show a qualitatively and significantly higher level of agreement with a ‘very good’ kappa value of 0.91 (95% CI, 0.86–0.95), even when they have started in an unplanned manner (kappa value of 0.85 (95% CI, 0.75–0.95)). In fact, the EP-planned group shows the higher kappa value: 0.93 (95% CI, 0.89–0.98). In summary, the patients who followed the EP changed their mind significantly less often than those who did not follow it, even though they started treatment unplanned. Nonetheless, there may be other factors driving this high concordance level, suggesting that not only are patients making firm and reflective choices but also the team in charge (and probably the whole health system) is also respecting those decisions.

Regarding CT, the treatment start date is considered to be when the choice was confirmed to be firm by the patients and/or their relatives.

The distribution of definitive techniques could be a marker of the degree of implementation of patients’ free choice. In our opinion, the closer the dialysis modality distribution is to 50%, the closer we come to free choice; we postulate that the values that lead the decision may have a normal distribution in the population, although it has not been demonstrated except for in anecdotal experiences [14,25,28].

A systematic review of qualitative studies on patient views on the most important aspects in RRT choice has emphasized the need for patients to consider their lifestyle preferences. This factor may be even more important than the medical consequences of each specific treatment [30]. Thus, patients with CKD base their choice on their own perception of which treatments cause the least impact on their lifestyle [18,30–31].

PDAs should follow international standards [29,32–36] to increase patients’ knowledge about available options, allow them to foresee how the treatments will affect their lives, increase their participation in the choice and reduce the conflict and anxiety involved in the decision. They should be used within a systematic process, and in addition to providing balanced information about the options, they should also allow patients to identify their own values, facilitating deliberation and communication and using simple language with information based on scientific evidence [32–33]. Additionally, to implement an EP, the following aspects must be covered: the design of clinical paths assuring that ALL patients are referred to the educator at the right moment, assigning skilled staff, and spending sufficient time on education. Finally, the patients, in the absence of clinical or social contraindications, will freely choose the modality that best fits their lifestyles [5,30–31], thereby complying with the law and best practices [2].

The PDAs used in our experience were previously validated in Spain [S2 File. Patient Decision Aid Validation Poster Oviedo (Spain) 2012], and they were reviewed and endorsed by patient associations and scientific renal societies. Initially based on educational materials developed by Baxter Healthcare, they were adapted in 2009 after being tested with patients and HCP and following the IPDAS recommendations, including the addition of new materials.

The moment when the choice is firm is patient-dependent. On average, patients made a decision after 2.6 visits, which suggests that some patients considered they did not need further reflection, because they skipped the deliberation phase, and that they already solved their questions in previous steps.

Approximately 45% of the patients started unplanned dialysis and, as expected, with a higher use of temporary vascular access and permanent catheters versus the planned group (36.5% and 24.8% vs. 0% and 9.9%, respectively). Therefore, less than 50% of the HD population started with an arteriovenous fistula, a similar percentage to that reported by Goldstein [35], despite multidisciplinary CKD care.

In our experience, 1 of every 3 educated patients started unplanned dialysis. This registry did not aim to explain the reasons why EP patients started as unplanned. Similarly, in a study by Buck [12], 33% of the entries were patients with previous nephrology care and, theoretically, with enough time for a planned start, although ultimately with an acute dialysis initiation. This is certainly an area for improvement. However, in our case 98.7% of the non-educated patients were unplanned. The registry did not include the reasons why some patients did not pass through the EP or the comorbidities or other medical factors. Thus, we ignore whether the unplanned start was the reason why the patients were not educated, or conversely, the result of a lack of education is the cause for more arbitrary patient care. Nevertheless, an unplanned start should not imply a lack of education, and a major “leakage" in the non-educated group was avoided because of the mandatory informed consent in Spain.

Most of the unplanned patients began with HD. There is a certain trend to maintain the status quo [30]. However, clinical outcomes published by a number of groups are at least equally satisfactory with PD as the acute start modality [36].

One of the main limitations of this registry is that it is not a randomized trial; thus, although there may be a causal relationship, we cannot attribute the results to the use of PDAs separately. Other aspects may have also contributed (the time spent, HCP’s attitude, patients’ comorbidities, cognitive status, etc.). Furthermore, there might be other factors for a “successful choice”, such as face-to-face educational sessions, patient group sessions, and visits to the HD and PD units. However, given the elicit-provide-elicit process that this EP follows as well as the standardization of the EP itself, we are confident that the provided information is comprehensive, understandable, realistic and appropriate. Additionally, we have little data on the characteristics of the hospitals that decided to use the PDAs; however, they are centers that are sensitive to the concept of patients’ free choice, which is supported by the small percentage of non-EP patients in this experience. As such, there might be bias in the association of the use of PDAs with the resulting distribution of RRT modalities. Moreover, we should expect some variation according to patient preferences center by center. However, many of the HCPs participating in this registry have passed through training sessions on communication skills delivered by experts from the Spanish Transplant Organization (ONT), seeking more effectiveness and avoiding bias when providing information.

The guidelines suggest therapeutic objectives and, to a lesser degree, quality indicators. It would be useful to establish and measure quality indicators for the EP in CKD units and to standardize the implementation of EP to facilitate active choice by the patients [8].

Finally, given the continuously increasing burden of end-stage renal disease, the implementation of this type of educational process not only benefits patients by allowing them to choose the treatment that best fits their values and preferences but also contributes to the sustainability of the health systems. Despite the similar clinical outcomes, there is an economic benefit associated with PD [37]. Thus, all the patients who chose PD in this experience have contributed to make RRT less costly in our country [38].

## Conclusions

In our experience and recognizing certain weaknesses of the study—including the lack of randomization and the failure to collect data on comorbidity, the mental state or the cognitive status of the patients in each group—the implementation, of a formal education process for shared decision-making with PDAs results in a balanced choice between PD and HD, with a slight increase of the preemptive TX choice compared with the results in the national registry. This study identified a high correlation between the choice and the start of treatment when using these PDAs. Furthermore, the patients who followed the EP changed their mind significantly less often than those who did not follow it, despite starting unplanned. The patients who did not go through this EP had a much lower index of choosing PD and changed their decision more frequently when starting the definitive treatment. In our opinion, a definitive modality in which the patient is included may be influenced to a greater extent by the EP than on a planned or an unplanned start. Therefore, and with the expressed reservations, we would recommend the education process with PDAs as a useful tool for a more balanced decision on the dialysis technique maintained over time.

## Supporting Information

S1 File Components of the Decision Aid Tools and Activities. (PDF)

S2 File Patient Decision Aid Validation Poster Oviedo (Spain) 2012. (PDF)

## References

[1] Law 41/2002, regulatory base of patient autonomy and of rights and obligations with regard to clinical information and documentation. B.O.E. number 274, November 15, 2002.

[2] Covic A, Bammens B, Lobbedez T, Segall L, Heimbürger O, van Biesen W, et al Educating end-stage renal disease patients on dialysis modality choice: a clinical advice from the European Renal Best Practice (ERBP) Advisory Board. NDT Plus 2010; 3: 225–233. 28657058 10.1093/ndtplus/sfq059 PMC5477971

[3] Pastor JL, Julian JC. Keys of the process of information and choice of modality of dialysis in patients with chronic kidney failure (Spanish). Nefrologia 2010; 1 (Suppl 1): 1520.

[4] Marrón B, Martínez Ocaña JC, Salgueira M, Barril G, Lamas JM, Martín M, et al Analysis of patient flow into dialysis: role of education in choice of dialysis modality. Perit Dial Int 2005; 25 (Suppl 3): S56–S59. 16048258

[5] Celadilla O, Julve M, Vives A, De Miguel M, Arribas MJ, Cagigal D. Evaluation of the information received by the patient who begins unplanned dialysis or coming from a transplant. Rev Soc Esp Nefrol 2007; 10 (3): 38–45.

[6] Gomez CG, Valido P, Celadilla O, Bernaldo de Quiros AG, Mojon M. Validity of a standard information protocol provided to end-stage renal disease patients and its effect on treatment choice. Perit Dial Int 1999; 19(5): 471–477. 11379861

[7] Finkelstein FO, Story K, Firanek C, Barre P, Takano T, Soroka S, et al Perceived knowledge among patients cared for by nephrologists about chronic kidney disease and end stage renal disease therapies. Kidney Int 2008; 74:1178–1184. 10.1038/ki.2008.376 18668024

[8] Prieto-Velasco M, Isnard Bagnis C, Dean J, Goovaerts T, Melander S, Mooney A, et al Predialysis education in practice: a questionnaire survey of centres with established programmes. BMC Research Notes 2014;7: 730–738 10.1186/1756-0500-7-730 25326141 PMC4210595

[9] Isnard Bagnis C, Crepaldi C, Dean J, Goovaerts T, Melander S, Nilsson EL, et al Quality standards for predialysis education: results from a consensus conference. Nephrol Dial Transplant 2014; 2014 Jun 23. pii: gfu225. [Epub ahead of print]. 10.1093/ndt/gfu225 PMC4479667 24957808

[10] Goovaerts T, Bagnis Isnard C, Crepaldi C, Dean J, Melander S, Mooney A, et al Continuing education: preparing patients to choose a renal replacement therapy. J Renal Care 2015 1 16: 10.1111/jorc.12106 [Epub ahead of print]. PMC7328707 25597792

[11] ERA-EDTA Registry. Annual Report 2011. Available: http://www.era-edta-reg.org/files/annualreports/pdf/AnnRep2011.pdf.

[12] Buck J, Baker R, Cannab AM, Nicholson S, Peters J, Warwick G. Why do patients known to renal services still undergo urgent dialysis initiation? A cross-sectional survey. Nephrol Dial Transplant 2007; 22: 3240–3245. 17616535 10.1093/ndt/gfm387

[13] Goovaerts T, Jadoul M, Goffin E. Influence of a pre-dialysis education programme (PDEP) on the mode of renal replacement therapy. Nephrol Dial Transplant 2005;20: 1842–1847. 15919693 10.1093/ndt/gfh905

[14] Liebman SE, Bushinsky DA, Dolan JG, Veazie P. Differences between dialysis modality choice and initiation. Am J Kidney Dis 2012; 59: 550–557. 10.1053/j.ajkd.2011.11.040 22305859 PMC3640994

[15] Cooper BA, Branley P, Bulfone L, Collins JF, Craig JC, Fraenkel MB, et al IDEAL Study. A Randomized, controlled trial of early versus late initiation of dialysis. N Engl J Med 2010; 363: 609–619. 10.1056/NEJMoa1000552 20581422

[16] Robinski et al The Choice of Renal Replacement Therapy (CORETH) project: study design and methods. Clin Kidney J. Advance Access published October 29, 2014. 10.1093/ckj/sfu111 PMC4389146 25859375

[17] McLaughlin K, Jones H, VanderStraeten C, Mills C, Visser M, Taub K, et al Why do patients choose selfcare dialysis? Nephrol Dial Transplant 2008; 23: 3972–3976. 10.1093/ndt/gfn359 18577531

[18] Davies SJ, Van Biesen W, Nicholas J, Lameire N. Integrated care. Perit Dial Int 2001; 21 (Suppl 3): S269–S274. 11887834

[19] Manns BJ, Taub K, Vanderstraeten C, Jones H, Mills C, Visser M, et al The impact of education on chronic kidney disease patients’ plans to initiate dialysis with selfcare dialysis: a randomized trial. Kidney Int 2005; 68: 1777–1783. 16164654 10.1111/j.1523-1755.2005.00594.x

[20] Levin A, Lewis M, Mortiboy P, Faber S, Hare I, Porter EC, et al Multidisciplinary predialysis programs: quantification and limitations of their impact on patient outcomes in two Canadian settings. Am J Kidney Dis 1997; 29:533–540. 9100041 10.1016/s0272-6386(97)90334-6

[21] Mason J, Khunti K, Stone M, Farooqi A, Carr S. Educational interventions in kidney disease care: a systematic review of randomized trials. Am J Kidney Dis 2008;51:933–951. 10.1053/j.ajkd.2008.01.024 18440681

[22] Saggi SJ, Allon M, Bernardini J, Kalantar-Zadeh K, Shaffer R, Mehrotra R. Dialysis Advisory Group of the American Society of Nephrology: considerations in the optimal preparation of patients for dialysis. Nat Nephrol Mag 2012; 8:381–389. 10.1038/nrneph.2012.66 22487703

[23] Demoulin N, Beguin C, Labriola L, Jadoul M. Preparing renal replacement therapy in stage 4 CKD patients referred to nephrologists: a difficult balance between futility and insufficiency. a cohort study of 386 patients followed in Brussels. Nephrol Dial Transplant 2011; 26: 220–226. 10.1093/ndt/gfq372 20610526

[24] Wu IW, Wang SY, Hsu KH, Lee CC, Sun CY, Tsai CJ, et al Multidisciplinary predialysis education decreases the incidence of dialysis and reduces mortality—a controlled cohort study based on the NKF/DOQI guidelines. Nephrol Dial Transplant. 2009;24:3426–3433. 10.1093/ndt/gfp259 19491379

[25] Prichard SS. Treatment Modality Choice in 150 consecutive patients starting ESRD therapy. Perit Dial Int 1996; 16: 69–72. 8616177

[26] Blake PG, Quinn RR, Oliver MJ. Peritoneal Dialysis and the Process of Modality Selection. Perit Dial Int 2013; 33 (3): 233–241. 10.3747/pdi.2012.00119 23660605 PMC3649891

[27] Korevaar JC, Feith GW, Dekker FW, van Manen JG, Boeschoten EW, Bossuyt PM, et al NECOSAD Study Group. Effect of starting with hemodialysis compared with peritoneal dialysis in patients new on dialysis treatment: a randomized controlled trial. Kidney Int 2003; 64: 2222–2228. 14633146 10.1046/j.1523-1755.2003.00321.x

[28] Winterbottom AE, Bekker HL, Conner M, Mooney AF. Patient stories about their dialysis experiences biases other´s choices regardless of doctor´s advice: an experimental study. Nephrol Dial Transplant 2012; 27: 325–331. 10.1093/ndt/gfr266 21642512

[29] 2012 Update of the IPDAS Collaboration Background Document. CHAPTER L: Establishing the Effectiveness. Available: http://ipdas.ohri.ca/resources.html.

[30] Morton RL, Snelling P, Webster AC, Rose J, Masterson R, Johnson DW, et al Dialysis modality preference of patients with CKD and family caregivers: a discrete-choice study. Am J Kidney Dis 2012; 60:102–111. 10.1053/j.ajkd.2011.12.030 22417786

[31] Morton RL, Tong A, Howard K, Snelling P, Webster AC. Patient views about treatment of stage 5 CKD: a qualitative analysis of semistructured interviews. Am J Kidney Dis 2010; 55: 431–440. 10.1053/j.ajkd.2009.11.011 20116914

[32] International Patient Decision Aid Standards (IPDAS) Collaboration reaches consensus on indicators for judging the quality of Patient Decision Aids presented at the 27th Annual Meeting of the Society for Medical Decision Making (October 21–24, 2005) San Francisco, CA, USA. Available: http://ipdas.ohri.ca/resources.html.

[33] O’Connor A, Llewellyn Thomas H. Stacey D. IPDAS Collaboration Background Document. International Patient Decision Aid Standards (IPDAS) Collaboration 2006 Available: http://ipdas.ohri.ca/resources.html.

[34] Stacey D, Légaré F, Col NF, Bennett CL, Barry MJ, Eden KB, et al Decision aids for people facing health treatment or screening decisions (Review). Cochrane Database Syst Rev. 2009 (3):CD001431 10.1002/14651858.CD001431.pub2 19588325

[35] Goldstein M, Yassa T, Dacouris N, McFarlane P. Multidisciplinary predialysis care and morbidity and mortality of patients on dialysis. Am J Kidney Dis 2004; 44: 706–714. 15384022

[36] Koch M, Kohnle M, Trapp R, Haastert B, Rump LC, Aker S. Comparable outcome of acute unplanned peritoneal dialysis and haemodialysis. Nephrol Dial Transplant 2012; 27: 375–380. 10.1093/ndt/gfr262 21622993

[37] Liu F, Gao X, Inglese G, Chuengsaman P, Pecoits-Filho R, Yu A. A global overview of the impact of peritoneal dialysis first or favored policies: an opinion. Perit Dial Int 2014 7 31. pii: pdi.2013.00204. [Epub ahead of print] 10.3747/pdi.2013.00204 PMC4520723 25082840

[38] Villa G, Fernández–Ortiz L, Cuervo J, Rebollo P, Selgas R, González T, et al Cost-effectiveness analysis of the Spanish renal replacement therapy program. Perit Dial Int 2012;32:192–199 10.3747/pdi.2011.00037 21965620 PMC3525412

