# Supplementary material for: The Concordance between Patients’ Renal Replacement Therapy Choice and Definitive Modality: Is It a Utopia?
Source: PLoS One. 2015 Oct 14;10(10):e0138811. doi: 10.1371/journal.pone.0138811 (PMC4605797; doi:10.1371/journal.pone.0138811)
Supplement: S1 File — (PDF) [file pone.0138811.s001.pdf]

Components of the Decision Aid Tools and Activities

Materials and Activities	Source of the Materials	Content	Type of Material	Location	Values Phase	Informative Phase	Deliberation Phase	Decision-Making Phase
**Flipchart** (paper format or PowerPoint presentation)	Adapted from an educational material developed by Baxter Healthcare	Contains information for the patient, a facilitation guide for the professional and elements of deliberation	Informative / Deliberation	In hospital with professionals and, preferably, a family member	Yes (only the general information about Kidney Disease, symptoms, emotions, etc.)	Yes (only the part corresponding to RRT modalities and CT)		
**Values cards** (paper or laminated format)	Initially based on an educational material developed by Baxter Healthcare but they were extensively modified as per the participants experience with their own patients	Cards that show affirmations that the educator puts on the table. The patients then interpret how important or unimportant they are based on their own personal values, selecting those which they consider most important.	Determining Values and Lifestyle	In hospital with professionals and, preferably, a family member	Yes		Only if the educator suspects changes in the values for some reason.	
**Decision-making software** (questionnaire in electronic format)	Locally produced based on the participants experience with their own patients	Questionnaire of 18 affirmations related to values, lifestyle and worries to which the patient assigns points (from 1 to 5) according to how strongly they agree with each affirmation and which calculates if their preference is for treatments at home or in the hospital based on a matrix.	Determining Values and Lifestyle	In hospital with professionals and, preferably, a family member	Yes		Only if significant lifestyle changes have occurred	

Materials and Activities	Source of the Materials	Content	Type of Material	Location	Values Phase	Informative Phase	Deliberation Phase	Decision-Making Phase
**Schedule of a work day and a normal weekend** (electronic format)	Locally produced based on the participants experience with their own patients	Time slots in which patients distribute the activities that they normally conduct. Helps patients anticipate how the treatment options could be adapted to their daily life.	Determining Values and Lifestyle	In hospital with professionals and, preferably, a family member	Yes		Only if significant lifestyle changes have occurred	
**Abbreviated brochure about dialysis modalities** (paper format)	Locally produced based on the participants experience with their own patients	Basic information about dialysis modalities that is used for pre-reading at home to facilitate understanding of the information that the health professionals will provide in the hospital.	Informative	At home with relatives and/or friends		Yes		
**Informational brochure for relatives and/or friends** (paper format)	Locally produced based on the participants experience with their own patients	Describes the decision process and how they can help the patients during this process.	Informative	At home with relatives and/or friends		Yes		
**DVD**	Locally produced with the participation of real Spanish patients and the Spanish Patient Association (ALCER)	Information about the treatments and images of real patients in different RRT options.	Informative / Deliberation	At home with relatives and/or friends, or in hospital		Yes	Yes	
**Expanded brochure about kidney disease, RRT modalities and CT** (paper format)	Adapted from an educational material developed by Baxter Healthcare	Contains all the information that the professional has provided the patient in the hospital during the informational phase.	Informative	At home with relatives and/or friends		Yes		

Materials and Activities	Source of the Materials	Content	Type of Material	Location	Values Phase	Informative Phase	Deliberation Phase	Decision-Making Phase
**Brochure about treatment planning for patients who are candidates for kidney transplantation** (paper format)	Locally produced based on the participants experience with their own patients	Contains specific information for patients who are potential candidates for transplantation from living and/or cadaver donors on the importance of planning their long-term treatment.	Informative	In hospital with professionals and, preferably, a family member		Yes		
**Brochure about social assistance benefits (paper format)**	Locally produced based on the experience of the Spanish Patient Association (ALCER)	Social assistance benefits to those patients in Spain who are eligible, in order to reduce the anxiety that these issues cause, which can alter the educational process.	Informative	At home with relatives and/or friends		Yes		
**Advantages and points to consider about dialysis modalities form** (paper format)	Adapted from an educational material developed by Baxter Healthcare	Patients record the advantages and disadvantages they perceive in each modality. The patients should then select those to which they give more weight.	Deliberation	In hospital with professionals and, preferably, a family member			Yes	
**Other Activities**	As per hospital protocol and regulation	Additional activities that the hospital implements according to its organization (visits to the PD and HD units, interviews with other patients, patient groups, etc.).	Deliberation	In hospital with professionals and, preferably, a family member			Yes	

Materials and Activities	Source of the Materials	Content	Type of Material	Location	Values Phase	Informative Phase	Deliberation Phase	Decision-Making Phase
**Situation form regarding decision making** (paper format)	Locally produced based on the participants experience with their own patients	Questionnaire that allows patients to evaluate in which phase in the decision process they are (if they are still evaluating the options, if they have an initial choice, or if the choice is definitive). The questionnaire is used from the beginning of the informational phase and thereafter, every time the patient visits the hospital.	Deliberation	In hospital with professionals and, preferably, a family member		Yes	Yes	Yes. Patients are considered to have made a decision when they report that they have made a definitive decision and are not going to change it.
**Informed Consent**	As per hospital protocol and regulation		Official Documents					Official consent forms from each hospital or autonomous region are used for the schoice, start of treatment, and performance of vascular or peritoneal access.
