# Supplementary material for: The Concordance between Patients’ Renal Replacement Therapy Choice and Definitive Modality: Is It a Utopia?
Source: PLoS One. 2015 Oct 14;10(10):e0138811. doi: 10.1371/journal.pone.0138811 (PMC4605797; doi:10.1371/journal.pone.0138811)
Supplement: S2 File — (PDF) [file pone.0138811.s002.pdf]

VIII Reunión Nacional
Diálisis Peritoneal
OVIEDO
2 al 4 de Febrero 2012

Valoración multicéntrica de un proceso educacional con Nuevas Herramientas de Ayuda para la Toma de Decisión Compartida del TSR

Escobedo Rumoroso, JM - Consorci Hospital General de Valencia
en nombre del Grupo de Valoración del Proceso Educacional con HATD del TSR*

INTRODUCCIÓN

- La elección del TSR es un derecho recogido en la ley
- Implica una toma de decisión crítica en situaciones clínicas y psicológicas que pueden distar de las ideales
- La HD y la DP son modalidades de tratamiento complementarias y no competitivas dentro de un tratamiento integrado de ERCA
- La información y educación que llega a estos pacientes es insuficiente.

Desarrolladas a raíz de la colaboración entre Baxter y 13 hospitales, las Herramientas de Ayuda a la Toma de Decisión (HATD) han sido avaladas por **ALCER, ONT, SEN, SEDEN, SAN, ADER, Federación Andaluza ALCER y ERTE**, y pretenden **facilitar una toma de decisión coherente con el estilo de vida y los valores del paciente.**

OBJETIVOS

Valoración descriptiva, prospectiva y multicéntrica para conocer la utilidad de las HATD dentro de un proceso educativo en modalidades de TSR para facilitar que el paciente realice una elección informada de su tratamiento.

MÉTODOS

Se utilizaron dentro de un **proceso educativo estructurado** en 4 fases:

1. Fase de identificación de los valores y estilo de vida
2. Fase informativa sobre la ERC y las modalidades de TSR
3. Fase de deliberación y resolución de dudas
4. Fase de toma de decisión.

Inicialmente se evaluó la **satisfacción de pacientes, familiares y adultos sanos con las HATD y el proceso de educación** con entrevistas telefónicas realizadas por agencia externa a 40 participantes (22 pacientes, 10 familiares y 8 adultos sanos). Se realizaron 46 entrevistas (28 de ellas a 22 pacientes). Posteriormente se realizaron entrevistas adicionales (7 a pacientes mayores y 6 a pacientes candidatos a Tx) para evaluar la satisfacción con materiales desarrollados específicamente para estos perfiles de pacientes, a petición de los profesionales.

RESULTADOS

- El 95% encuestados afirmó que los materiales, incluyendo los específicos que ayudan al paciente a identificar sus valores y estilo de vida, les habían ayudado a comprender los tratamientos y clarificar sus alternativas y sus preferencias.
- Un 82% y un 95% respectivamente valoró como adecuada/muy adecuada en cantidad y claridad la información.
- Un 65% de los encuestados era capaz de describir las distintas técnicas con sus propias palabras tras su uso.
- La intención inicial de elección de TSR fue de un 67% para la DP, un 23% para la HD y 10% ns/nc.
- Todos los familiares manifestaron tener un papel activo en la elección del tratamiento como asesores (60%) o como decisores únicos o parciales del tratamiento.
- Todos los pacientes mayores y los pacientes candidatos a Tx manifestaron que los materiales aclaraban sus dudas.
- También se realizaron entrevistas independientes a profesionales sanitarios. Tanto pacientes como profesionales confirmaron su satisfacción con el proceso de educación y con las HATD.

Pacientes (Informados y No Informados)	Edad media	% de la muestra	% Incidencia TSR según Registro SEN 2007
Total (N=22)	60,2	100%	
< 65 años (N=1)	53,7	64%	45%
≥ 65 años (N=8)	72,3	36%	55%
**Mayores** (N=7)	76,0	n/a	
**Candidatos a Tx** (N=6)	45,0	n/a	

Distribución de edades medias de los 2 grupos de pacientes iniciales y comparación con la incidencia de TSR publicada por la SEN, y edades medias de los grupos de pacientes mayores y candidatos a Tx

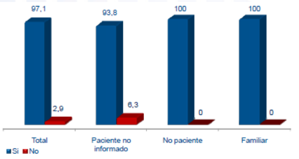

Grado de Utilidad de los Materiales Informativos

Elección por rango de edad	Edad media	Intención Elección	% sobre el total
DP < 65 años	53,0	11	79%
HD < 65 años	56,0	3	21%
DP ≥ 65 años	75,5	3	38%
HD ≥ 65 años	73,7	3	38%
NS/NC ≥ 65 años	67,0	2	25%

Intención de elección de la modalidad de diálisis por rangos de edad

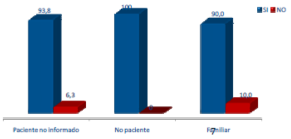

Capacidad para hacer comprender los tratamientos

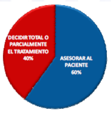

Papel del familiar en la toma de decisión

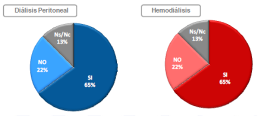

Capacidad del paciente para explicar las opciones con sus propias palabras

CONCLUSIONES

- Estas nuevas HATD utilizadas en el marco de un proceso de educación estructurado confirmarían su utilidad proporcionando información y clarificando y facilitando la elección informada del TSR basada en los valores del paciente.
- Esta aproximación ha sido positivamente valorada por pacientes, familiares y profesionales.
- La implementación de un proceso educativo estructurado con estas HATD podría también derivar en un incremento de la elección de DP.

*Grupo de Valoración del Proceso Educacional con HATD del TSR

Consorci Hospital General de Valencia: Escobedo JM, Galán A, Martí A, Martínez JF
Complejo Hospitalario Universitario de A Coruña: Aneiros R, Bermúdez C, Mojón M, Pérez T, Pérez-Fontán. M, Portela L, Rodríguez-Carmona A
Complejo Asistencial Universitario de León: Aguilera AI, Granda M, Prieto M
Complexo Hospitalario de Ourense: Novoa E, Otero A. Rodríguez T
Fundación Renal ALCER España: Bogeat J, Julián JC
Hospital Clínico Universitario de Valencia: González M
Hospital Comarcal d'Inca: Albert J
Hospital General de Castelló: Cerrillo V, Fenollosa MA, Renau E, Villatoro J
Hospital Infanta Sofía, Madrid: de Álvaro F, Aragoncillo I, Cirugeda A, Hevia C, Reyes MP
Hospital Son Llàtzer, Palma de Mallorca: Buades JM
Hospital Universitari Arnau de Vilanova, Lleida: Craver L, Fernández E, Gutiérrez JM, Vilagrasa E.
Hospital Universitario Fundación Alcorcón: Hernández ME, Portolés JM
Hospital Universitario de Puerto Real: Benavides B, Quirós PL, Remón C, Tejuca A
